# Unexplained Anemia in the Elderly

**DOI:** 10.7759/cureus.19971

**Published:** 2021-11-28

**Authors:** Jose C Alvarez-Payares, Sebastián Rivera-Arismendy, Pablo Ruiz-Bravo, Sara M Sánchez-Salazar, Rene A Manzur, Sara I Ramirez-Urrea, Andres Puello

**Affiliations:** 1 Internal Medicine, University of Antioquia, Medellín, COL; 2 Internal Medicine, University of Antioquía, Medellín, COL; 3 General Medicine, Fundación Universitaria San Martín, Medellín, COL; 4 Internal Medicine, University of Sucre, Sincelejo, COL

**Keywords:** clonal cytopenia, myelodysplastic syndromes, chronic inflammatory diseases, elderly patient, anemia

## Abstract

Anemia is frequently diagnosed in elderly patients, and it is a key indicator of many reactive and clonal conditions. Furthermore, the older age is the most common presenting age for myelodysplastic syndromes (MDS). Anemia in older age may be attributed to an inflammatory state due to senescence, comorbidities, nutritional deficiencies, or primary bone marrow conditions. As diagnostic possibilities and life expectancy increase, the prevalence of anemia of the elderly increases as well. The etiology has a direct impact on the treatment and quality of life of these patients, in whom is a usual clinical challenge as it may be due to a multifactorial origin. In a minority group, when no etiology is identified, it is classified as unexplained anemia (UA) or clonal cytopenia of unknown significance (CCUS). The underlying cause of anemia remains unexplained in 30% of cases, and a great part of unexplained cytopenia may account for myeloid neoplasms. Anemia in the elderly is associated with worse cognitive and functional outcomes and increased mortality.

## Introduction and background

Anemia is a common condition in elderly age, with a prevalence up to 17% in patients older than 65 years [1]; in addition, its prevalence has increased with higher life expectancy and better diagnostic tests. In older patients, it is more frequently associated with gastrointestinal (GI) diseases, chronic kidney disease (CKD), cancer, and myelodysplastic syndrome (MDS). Due to elder age and its own setting, different causes may add up and promote anemia development, generating a diagnostic and treatment challenge [2].

According to its etiology, anemias may be classified as nutritional deficiency anemia, bleeding anemia, clonal anemia, and secondary to chronic inflammatory diseases (including CKD).

Identifying the cause is paramount to offer a specific and efficient therapy for this population. When no cause is identified, patients were previously classified to have unexplained anemia (UA), as was described by Artz et al. [3]. Nonetheless, recent classifications based on bone marrow (BM) studies suggest that it must be classified as idiopathic cytopenia of unknown significance (ICUS) with isolated anemia (ICUS-A) [4]. Furthermore, a condition known as clonal cytopenia of unknown significance (CCUS) has been described, which consists of cytopenic patients with somatic mutations in peripheral blood leukocytes that do not meet diagnostic criteria for MDS or other neoplastic BM conditions [4].

In patients with unknown etiology or numerous comorbidities, anemia management requires a multidisciplinary approach that grants the understanding of the physiologic changes of elderly age. In most cases, nutritional supplementation or etiology removal would be enough; however, long-term treatment with directed therapy, continuous erythropoietin (EPO) therapy, or blood transfusions may be required. The main goal is a clear impact on the quality of life and safety of the patient [2]. In the present article, we review current concepts surrounding clinical relevance, pathogenesis, and management of UA on elderly patients.

## Review

Definition of anemia related to older age

Although hemoglobin levels decrease with age, we will assume the definition of anemia in accordance with the World Health Organization (WHO) criteria, which was defined as a hemoglobin concentration of <13.0 g/dL in men and <12.0 g/dL in women [5].

Epidemiology of anemia on elderly patients

**Figure 1:**
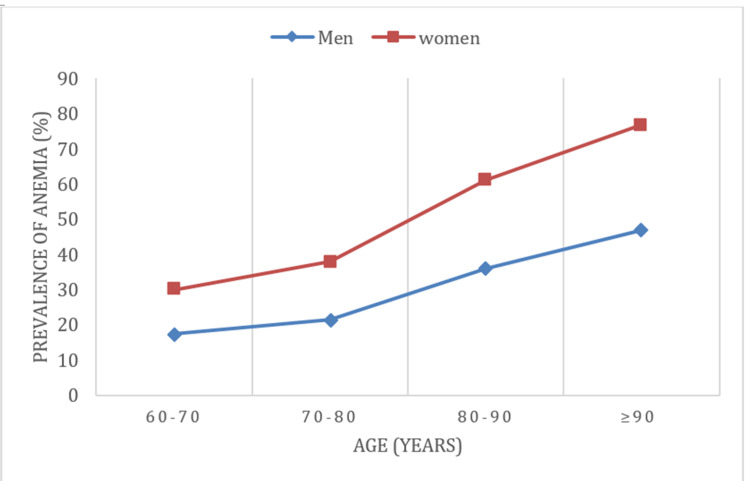
Increase of anemia prevalence in older adults Increase of anemia prevalence in patients with advanced age from a cohort of 19758 inpatient and outpatient visits from university hospitals (modified from Steensma et al. [6])

Large prospective studies have revealed a general prevalence of anemia of 10%-24% in older adults [5]. Anemia is one of the main conditions of elderly inpatients, with a prevalence of 40%, being most common in nursing homes with 47%. Prevalence increases with age, up to 50% in men older than 80 years, both inpatient and outpatient (Figure 1). An increase of such figures is expected due to longer aging associated with more diagnostic possibilities [5].

Clinical relevance of older age anemia

Anemia is associated with a wide spectrum of clinically relevant conditions; hence, identifying even mild anemia is crucial (Table 1). Low hemoglobin levels represent a risk factor for cardiovascular diseases [7], cognitive impairment [8], insomnia [9], mood disorders [10], worse quality of life [11,12], and impaired executive function and physical performance [11,13], with higher fall and fracture risk [12].

Anemia presence is associated with frequent hospital admissions [14] and longer inpatient stance [15]; therefore, it has been concluded that anemia is a mortality marker [16], with higher mortality risks when anemia is secondary to nutritional disorders or CKD; both may be observed in patients with UA.

**Table 1:** Distribution of anemia etiology in elderly patients (data from four studies [2,9,17,18]) CKD: chronic kidney disease; UAE: unexplained anemia of the elderly

Etiology	Frequency (%)			
Country	USA-1 [2]	USA-2 [17]	Italy [18]	Poland [9]
Iron deficiency	16.6	25.3	16.0	13.0
B12- and/or folate-deficiency anemia	14.3	<1	9.5	7.1
Iron- and B12- and/or folate-deficiency anemia	3.4	-	-	2.4
Chronic disease/inflammation anemia	19.7	9.8	17.4	33.1
CKD anemia	8.2	3.4	15.0	1.2
CKD and chronic disease/inflammation anemia	4.3	-	-	-
UAE	33.6	43.7	26.4	28.4
Clonal hematopoiesis	-	7.5	1.8	-
Others	-	10.3	14.4	14.8

Pathogenesis and basic mechanisms of anemia in elderly age

**Figure 2:**
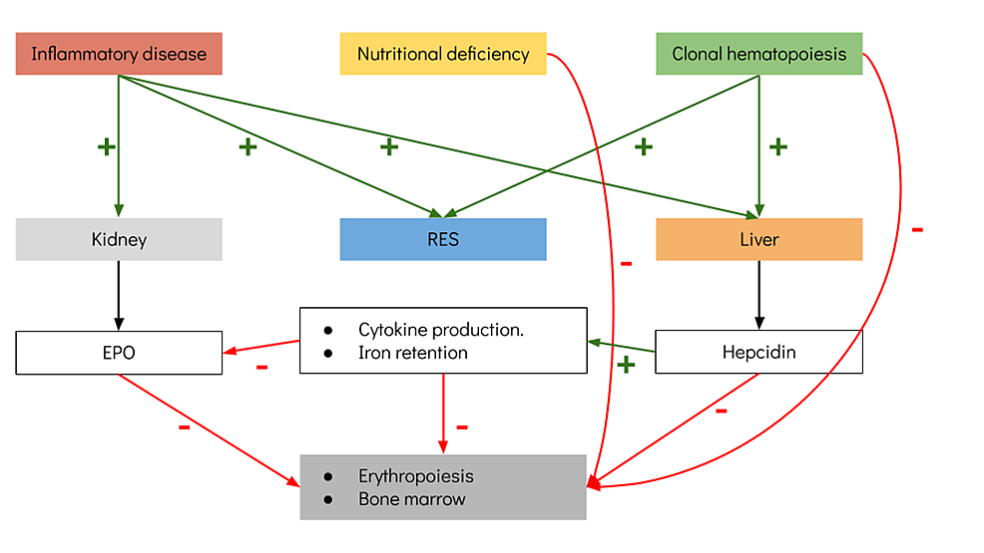
Possible mechanisms of anemia in elderly adults (Modified from Shlush [19])

Anemia in the elderly usually has multiple underlying causes and is frequently associated with more than one predisposing factor. Based on physiologic concepts, underlying diseases may be classified as inflammatory anemia (especially due to CKD), nutritional deficiency, and clonal hematopoiesis. Insufficient EPO production is remarkable, which is commonly observed in patients with CKD; however, it may be observed in patients with normal renal function, which is a key factor in patients with clonal cytopenia of unknown significance [6]. Patients with underlying inflammatory disorders may present an increased hepcidin hepatic production, which leads to decreased erythropoiesis and iron uptake in the reticuloendothelial system. Furthermore, EPO production is insufficient in response to anemia, with a mild impact of EPO on erythropoiesis (Figure 2). Another distinctive characteristic is increased phagocytosis of senescent red blood cells (eryptosis).

In the elderly patient with anemia, an initial workup to rule out GI bleeding and nutritional deficiency of common factors that could lead to anemia (B12, folate, iron) is mandatory. After this initial workup, it is essential to remember that one-third of patients with CKD or inflammatory anemia (related to cancer, autoimmune disease, or chronic infections) may present a pro-inflammatory state, with insufficient EPO production. This is directly related to increased hepcidin levels, an important acute-phase protein synthesized in the liver. Hepcidin presence leads to a reduction of iron intestinal absorption and reduced iron release by macrophages [19-21]. Patients with cancer, CKD, and autoimmune diseases and elderly adults may all have elevated hepcidin levels, attributed to a pro-inflammatory state or “inflamm-aging,” which is directly related to age. Many pro-inflammatory cytokines are increased in this state, such as IL-1, IL-6, and tumor necrosis factor-alpha (TNFa); conversely, a reduction of autophagy secondary to increased NF-κB signaling and increased reactive oxygen species (ROS) may lead to increased inflammasome response [20].

In such a pro-inflammatory state, it is paramount to identify whether or not it is due to physiologic changes related to aging or if it is evidence of systemic response of an unidentified condition. Slightly elevated IL-6 levels, which are caused by changes of body composition or underlying inflammatory disease, may inhibit EPO production and/or hepcidin activation. Difficulties in IL-6 detection limit the capabilities of routine assessment [20].

In elderly adults, clonal leukocytes have been detected, with somatic mutations increasing with age. This clonal hematopoiesis is related to a higher prevalence/mortality of hematologic malignancies such as MDS [22,23]. In healthy subjects without cytopenias, this condition is called clonal hematopoiesis of indeterminate potential (CHIP); however, once there is anemia, the diagnosis changes to clonal cytopenia of unknown significance (CCUS) or MDS, considering that many patients with CCUS progress to MDS (Table 2) [22,23]. Both CHIP and CCUS patients may develop other hematologic malignancies; hence, a leukocyte thorough study is recommended to establish whether or not somatic mutations are present in patients with unexplained cytopenias, as well as bone marrow plus flow cytometry evaluation to determine if cytogenetic anomalies are present [24].

**Table 2:** Classification of aging-related clonal hematopoiesis UA: unexplained anemia; ICUS: idiopathic cytopenia of undetermined significance; CCUS: clonal cytopenia of undetermined significance; CHIP: clonal hematopoiesis of indeterminate potential; MDS: myelodysplastic syndrome; nd: not detected

Condition	UA	ICUS	CCUS	CHIP	MDS
Cytopenia	+	+	+	-	+
Dysplasia	nd	-	-	-	+
Cytogenetic abnormalities	nd	-	-	-	+
Somatic mutations	nd	-	+	+	+

Patients with cytopenia without molecular abnormalities nor criteria for other diseases such as MDS will be included in the idiopathic cytopenia of unknown significance (ICUS) group and those with anemia in the idiopathic cytopenia of unknown significance with anemia (ICUS-A) group [24]. As ICUS-A prevalence is directly related to age, this has been considered as “anemia of older age” [25].

A reduction in EPO levels in the absence of CKD or any other inflammatory causes would lead to a possible ICUS-A, which could be explained by an intrinsic defect of EPO in the presence of an older kidney or a lower testosterone and estrogen production. It is still unclear whether it is possible or not for ICUS-A to present with an adequate EPO production [26]. Patients with MDS may also present with lower EPO production, which may respond to recombinant EPO treatment [26].

Diagnostic aspects

The basic anemia workup in elderly patients requires that the most common etiologies be ruled out through different diagnostic studies (Tables 3, 4). Each patient’s context will guide the need for additional studies such as upper gastrointestinal tract endoscopy or abdominal ultrasound. In the presence of additional anomalies or signs of clonal hematologic disease, a bone marrow biopsy and aspiration is mandatory to rule out hematologic diseases, including MDS [2]. As with many other conditions, anemia workup must be directed by the patient’s life expectancy; many authors consider that a bone marrow aspiration is adequate when life expectancy is at least three months [27].

**Table 3:** Laboratory tests on the elderly patient

Initial tests	Tests
Hematology	Complete blood count
Blood chemistry	Creatinine, blood urea nitrogen, glomerular filtration rate creatinine
Serum iron parameters	Iron, ferritin, total iron-binding capacity
Nutritional anemia parameters	Serum B12, red blood cell folate levels
Secondary tests	Tests
Hemolysis suspicion	Bilirubins, lactate dehydrogenase, reticulocyte count, direct antiglobulin test, peripheral blood smear, haptoglobin
Other tests in men	Serum testosterone
Other negative tests with macrocytosis and/or other cytopenias	Bone marrow examination or molecular profiling for clonal hematopoiesis
Isolated normocytic anemia with a reticulocyte count of 10000/μL or less	Bone marrow examination for pure red cell aplasia

**Table 4:** Laboratory findings of common anemias in elderly adults IDA: iron-deficiency anemia; UA: unexplained anemia; CDA: chronic disease anemia; CKD: chronic kidney disease; MDS: myelodysplastic syndrome; MCV: mean corpuscular volume; TIBC: total iron-binding capacity; ESR: erythrocyte sedimentation rate; CRP: C-reactive protein; EPO: erythropoietin; CrCl: creatinine clearance; TSH: thyroid-stimulating hormone; MMA: methylmalonic acid

Type of anemia	MCV	Iron/TIBC	Ferritin	ESR/CRP	EPO	CrCl	Albumin	Miscellaneous
IDA	Small	Low/high	Low	nl	High	nl	nl	
UA/CDA	Small	Low/high	Low/high	Alto	Bl	nl	Low-nl	
CKD	nl	nl	nl	nl	Low	<30 mL/minute	Low-nl	
Hypothyroidism	Large	nl	nl	nl	High	nl	nl	High TSH
B12/folate	Large	nl	nl	nl	High	nl	nl	Low vitamin levels, high MMA
MDS	Large	nl	nl	nl	High	nl	nl	Bone marrow study may be diagnostic
Desnutrition	nl	nl	nl	nl	Bl	nl	Low	
UA	nl	nl	nl	nl	Bl	nl	nl	

It is correct to assume that a patient may have a bone marrow clonal disorder if no concluding results are available after a molecular evaluation, cytogenetic studies, and/or flow cytometry are performed. The detection of clonal myeloid cells may cause a change in the diagnosis, i.e., from ICUS to CCUS or even MDS [27].

Management

No reports have been published describing the natural history of UA or if treatment may improve any of the adverse associations previously described. In UA, therapy is not usually indicated [28]. Anemia degree is usually modest; a severe/symptomatic anemia may lead to a diagnosis reassessment.

A study has demonstrated an improved hemoglobin concentration and quality of life in African-American elderly patients with chronic UA previously treated with EPO [17]; however, no proper studies have been designed to evaluate EPO therapy for UA patients.

A significant proportion of male patients with UA and low testosterone levels will improve their hemoglobin concentration after testosterone therapy [18]. Accordingly, a multicentric, randomized clinical trial was published, which evaluated testosterone treatment in elderly male patients with low testosterone levels at baseline [18]. Testosterone gel was administered with adjusted doses to maintain testosterone levels in a normal range for 12 months. The results showed that, from 788 patients, 126 were anemic and 62 were classified as having UA. More than 50% of anemic subjects had a hemoglobin increase of >1 g/dL at month 12 of treatment. Despite this encouraging result, investigators properly noted that no health benefit has been observed from a higher hemoglobin level [18].

Some patients with UA and normal ferritin (but lower than 200 ng/mL) may respond to intravenous iron [29]. In these patients, a previously undiagnosed iron-deficiency anemia must be assumed, and an extensive workup for a bleeding source is mandatory [29].

At the moment, different drugs with hypoxia-inducible factors, which may impact patients with low endogenous EPO levels, represent a future treatment option for UA in elderly patients [27].

## Conclusions

Anemia in older persons is a challenge in clinical practice. In many cases, various etiologies could be detected, and a meticulous investigation leads to the correct diagnosis. In a great proportion of patients, no underlying cause of anemia is found after the first evaluation, resulting in the provisional diagnosis of UA. Nevertheless, in plenty of cases, no underlying etiology is found even after a thorough diagnostic workup that includes an examination of different organ systems, including the bone marrow, and cytogenetic and molecular studies. In these kinds of patients, in many cases, only follow-up can be offered. Finally, it is difficult to determine which patients with unexplained anemia could closely develop hematological neoplasms, although at present there are already great advances in this regard.
